# User Experience Evaluation of Upper Limb Rehabilitation Robots: Implications for Design Optimization: A Pilot Study

**DOI:** 10.3390/s23219003

**Published:** 2023-11-06

**Authors:** Tzu-Ning Yeh, Li-Wei Chou

**Affiliations:** 1 Department of Medical Engineering and Rehabilitation Science, China Medical University, Taichung 404332, Taiwan; 106309202@365.cmu.edu.tw; 2 Department of Physical Medicine and Rehabilitation, China Medical University Hospital, Taichung 404332, Taiwan; 3 Department of Physical Therapy and Graduate Institute of Rehabilitation Science, China Medical University, Taichung 406040, Taiwan; 4 Department of Physical Medicine and Rehabilitation, Asia University Hospital, Asia University, Taichung 413505, Taiwan

**Keywords:** upper limb robots, rehabilitation, stroke, formative test

## Abstract

With the development of science and technology, people are trying to use robots to assist in stroke rehabilitation training. This study aims to analyze the result of the formative test to provide the orientation of upper limb rehabilitation robot design optimization. We invited 21 physical therapists (PTs) and eight occupational therapists (OTs) who had no experience operating any upper limb rehabilitation robots before, and 4 PTs and 1 OT who had experience operating upper limb rehabilitation robots. Data statistics use the Likert scale. The general group scored 3.5 for safety-related topics, while the experience group scored 4.5. In applicability-related questions, the main function score was 2.3 in the general group and 2.4 in the experience group; and the training trajectory score was 3.5 in the general group and 5.0 in the experience group. The overall ease of use score was 3.1 in the general group and 3.6 in the experience group. There was no statistical difference between the two groups. The methods to retouch the trajectory can be designed through the feedback collected in the formative test and gathering further detail in the next test. Further details about the smooth trajectory must be confirmed in the next test. The optimization of the recording process is also important to prevent users from making additional effort to know it well.

## 1. Introduction

Stroke has been one of the top ten causes of death in the past ten years in Taiwan [1] and usually leaves various sequelae, such as motion function disability, paresthesia, and cognitive dysfunction [2]. The sequelae of stroke not only affect patients’ future daily lives but also cause the occurrence of other diseases. With the development of science and technology, people try to use robots to assist in rehabilitation training [3,4]. The functions of rehabilitation robots include the assistance of training actions, the recording and analysis of rehabilitation training, and providing the environment for functional activities. Studies show that the effect of robot-assisted therapy has no statistical difference compared with conventional therapy [5,6]. Compared with conventional rehabilitation training, the advantages of robotic rehabilitation training include allowing the therapist to assist multiple patients’ training at the same time, recording and analyzing the patient’s training process, providing a reference for the formulation of training plans, and creating an environment closer to daily life for functional activities training.

Training for upper limbs is usually more diverse than lower limb training, and the time required for rehabilitation is usually longer. Therefore, we hope to study the technology of rehabilitation machines in the field of upper limb rehabilitation. There are many types of stroke upper limb rehabilitation robots on the market [7,8,9]. They can be divided into proximal and distal training robots according to training joints. Most of the proximal robots provide training for motions of shoulder joints, elbow joints, and pronation and supination [8], while the distal robots provide training for motions of the wrist joint and finger joints [10]. The contact between the robot and the limbs can be divided into exoskeleton [11] and end-effector robot [12]. The exoskeleton robot connects the user’s arm with multiple points. The rotation axes of robot joints usually coincide with human joints. The end-effector robot connects the user arm at the distal point only. One recent study shows that the end-effector robot is better than the exoskeleton with regard to participation and activity among chronic stroke patients [13], and another study shows that the exoskeleton robot is better than the end-effector robot in the treatment of finger motor impairment [14]. The range of training action space can be divided into 2D and 3D training space approaches. 2D rehabilitation robots usually come with a desktop to bear the patient’s arm weight, helping them to focus on practicing motion control [15]. Training activities are limited to those on the desktop. Robots with a 3D training range can provide motion closer to the activities in reality, and gravity compensation is usually provided by robot motors [16,17,18]. Though robot-assisted therapy is known to have the same effect as conventional therapy, it still has room for development and study. Along with the kinematic sensors being widely used in the robot-assisted system and the data analysis technique being mature, recent studies research deeper into the interaction between robots and humans, and the relationship between kinematic data and human activity by quantifying the performance of humans and robots.

It has been decades since people studying trajectory planning and smoothing commonly applied the technique to the trajectory planning of automated vehicles and industrial manipulators. The main target of the trajectory planning process is to generalize a path from the start to the endpoint with kinetic constraints. Studies develop path-planning methods and optimized algorithms that generate obstacle-avoiding and collision-avoiding trajectories, like roadmap technologies, cell decomposition algorithms, artificial potential methods, and retraction techniques [19]. The trajectory-planning methods focus on shorter paths, lower energy consumption, and other constrained kinetic conditions [20]. With the development stage comes to the practical application; the technologies of real-time trajectory updating [21,22] and noise tolerance [23,24] are developed to face the dynamic environment. Further optimization of trajectory planning is developed by applying artificial intelligence techniques like neural networks, evaluation algorithms, swarm intelligence, and fuzzy logic [25,26].

The formative test is commonly performed during the design process of medical devices to optimize their safety and ease of use [27]. A reliable formative test can reduce the required number of tests during development and make the design process much more efficient. The medical device usually goes through several formative tests before the usability test. As the pilot test of usability, the result of the formative test is crucial for the development team to provide orientations of optimization. The purpose of this study is to analyze the result of the formative test to provide the orientation of upper limb rehabilitation robot design optimization and to discuss the process of transferring qualitative feedback of questionnaire results to quantitative industrial specifications.

## 2. Materials and Methods

**Figure 1:**
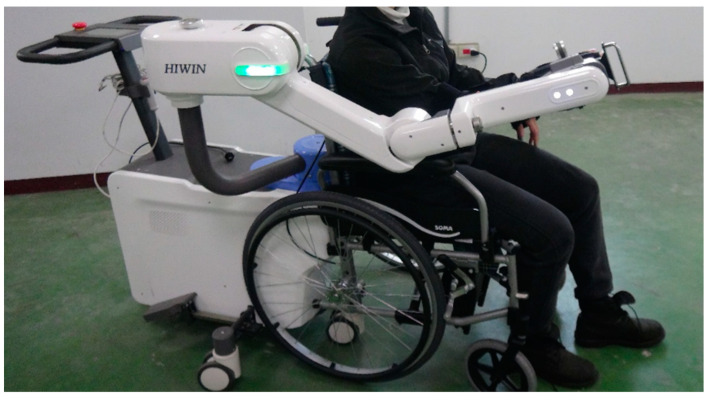
Upper limb rehabilitation robot U100 (permitted via HIWIN Technologies Corp., Taichung City, Taiwan).

U100 (Figure 1) is an exoskeleton robot designed by HIWIN Technologies Corp., Taichung City, Taiwan [28]. It supports the 3D motions in the range of motion of the shoulder, elbow, and wrist supination/pronation. The motion recording function is the main function of U100. The therapist leads the patient‘s arm by leading the exoskeleton arm and recording suitable training motions for them, and therefore is able to highly customize training plans. With the real-time gravity compensation function, the therapist can lead training motions without loading the weight of the patient’s arm and the exoskeleton. Another feature of U100 is spasticity protection [29]. When the robot detects abnormal torque and the risk of hazards, it will provide a reverse torque inversely proportional to the distance between the robot and the patient, creating a virtual wall between the robot and the patient.

The formative test in the study is the first out-of-plant test of U100. Considering that the device has not passed the usability test, we invited a standardized patient, who is a healthy subject, instead of a real patient, to ensure safety during the test. Also, asking the standardized patient to simulate the situation can reduce the variables among tests. The main subject of the formative test is to study the safety, the feasibility of the motion record function, and the ease of use of operations to figure out the optimized orientation of U100. As a pilot study of the clinical feasibility and the usability of the robot, we invited a small group of potential operators of U100 to join the test, including 21 physical therapists (PTs) and 8 occupational therapists (OTs) who have no experience in operating any upper limb rehabilitation robot before to participate in the test experiment as the general group. In addition, to compare the upper limb rehabilitation robots on the market, and to compare the opinions of therapists with different experiences with the rehabilitation robots, we also invited 4 PTs and 1 OT who have experience in operating upper limb rehabilitation robots as the experience group.

**Figure 2:**
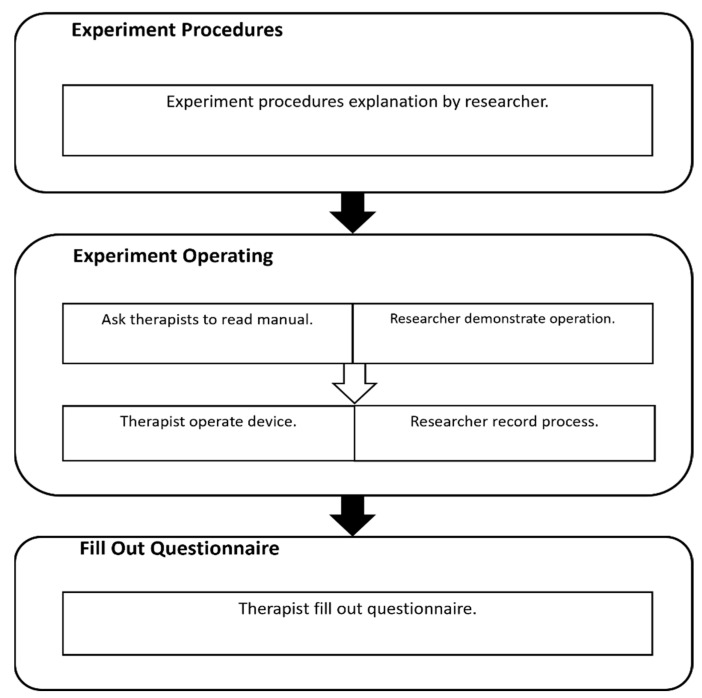
Experiment process.

The experiment process is shown in Figure 2. First, the operation method is demonstrated to the therapist, then a standardized patient is asked to simulate the situation in the treatment room, and finally, the therapist is asked to fill in a questionnaire in the interview. The demonstration takes about fifteen minutes; then, the therapist has fifteen minutes to practice the operation process. After the practice, the therapist will operate the device following the simulated situation, and it takes about thirty minutes. The standardized patient acts like a patient who has right hemiplegia 2 months after stroke and is Brunnstrom Stage II, and the setting environment is in an independent treatment room in the hospital, and there are only therapists, patients, and family members or caregivers in the room. Training tasks and function testing include passive range of motion (PROM), functional activities, troubleshooting procedures in emergencies, etc. There are two types of questionnaires: multiple-choice and open-ended questions (Supplementary materials). Participants fill out the questionnaire in an interview. The content of the questionnaire includes the safety of the rehabilitation robot for therapists and patients, the clinical applicability of the robot’s functions and the operational process, and the ease of use of the operational process. We also inquired about suggestions for the future development of the robot.

The Likert scale question, a psychometric scale, is one of the most widely used question types. It is easily summarized and is often used in a formative test [30,31,32,33]. In a Likert scale survey, respondents are asked how much they agree or disagree with a set of statements using a particular survey question instead of choosing between yes and no. To quantify the subjective opinion of therapists, we designed the questionnaire referring to the System Usability Scale (SUS). To know the usability of the robot compared to the conventional rehabilitation training, we planned the questions based on conventional training activities (Supplementary materials). The five-point agreement scale used to measure respondents’ agreement with various statements in our study has the advantage of obtaining more detailed opinions compared to numerical rating scales and is easy to understand and respond to. An average score of 3 points is considered to indicate that the robot has a similar function to conventional rehabilitation training and is applicable in clinical settings. Safety is recognized as safe with a score of 3 or more, and ease of use is recognized with a score of 3 to operate correctly without specific training or practice since the robot does not require passing an exam of operation before using it. Open-ended questions manually extract the number of people who are reflected in the statistical opinions after keyword classification and present them as a percentage of the total number of experimental people.

The demographic statistics of recruited therapists are shown in Table 1. The main clinical experience of PTs is in neurological and orthopedic physiotherapy fields, and the main clinical experience field of OTs is physiological functional therapy.

## 3. Results

The results for the multiple-choice questions are shown in Table 2 in the average score of all questions related to the topic. We use a two-tailed, two-sample equal variance (homoscedastic) *t*-test in Excel to analyze the difference between the two groups. For safety-related topics, the general group scored 3.5, and the experience group scored 4.5. Regardless of whether the therapist has experience in operating rehabilitation robots or not, the rehabilitation robot is considered safe for therapists. The experience group scored significantly higher than the general group. In applicability-related questions, the main function score was 2.3 in the general group and 2.4 in the experience group. The overall ease of use score was 3.1 in the general group and 3.6 in the experience group. There was no statistical difference between the two groups in applicability and ease of use topics.

The result shows that the record function has a low score of less than 3, which is not applicable in clinical. The reason for the evaluation is shown in the answers to open-ended questions, sorted out in Table 3.

The research on the optimization of the record function is discussed in the discussion section.

## 4. Discussion

Since the operating experience of different robots may influence the operation and the impression of ease of use, we will separately discuss the feedback of the experience group and the general group and analyze the subject in the experience group.

### 4.1. Experienced Group

Five therapists with robot operating experience were invited to participate in the experiment. The first subject is a PT having one to three years of clinical experience and mainly working in the orthopedic and neurology fields. The robot operating experience is a continuous passive motion machine. The safety of the device receives a score of 4. The motion record function receives a score of 3 in clinical applicability. The therapist thinks that the device replays the recorded trajectory. However, it needs a function to retouch the trajectory after recording since it is hard to record a smooth motion involving multiple joint movements without practice. Therefore, the therapist tends to use the device in orthopedic rehabilitation. If the device is going to be used for other purposes, like functional training, the therapist suggests the development team optimize the motion record function to provide a smooth trajectory, adding visual feedback and preventing the connection between the robot arm and the user’s arm to limit the motion of the distal limb. The ease of use of the device operation receives an average score of 4. The therapist thinks it is easy to learn and operate, and the instructions are easy to follow.

The second subject is an OT having one to three years of clinical experience and mainly working in the physiology field. The therapist has the robot operating experience of a self-made exoskeleton. The safety of the device receives a score of 4. The motion record function receives a score of 3 in clinical applicability. The therapist thinks that the device replays the recorded trajectory. However, it needs to provide the function of retouching the trajectory after recording since it is hard to record a smooth motion involving multiple joint movements without practice. If the device is going to be used for other purposes, like functional training, the therapist suggests the development team optimize the motion record function and add the assist and the resist training mode. The ease of use of the device operation receives an average score of 3.6 because the therapist thinks the changing side operation needs some practice.

The third subject is a PT having five to ten years of clinical experience mainly working in the orthopedic and neurology fields. The robot operating experience is ArmeoSpring from HOCOMA. The safety of the device receives a score of 4. The motion record function receives a score of 2 in clinical applicability. The therapist suggests the development team provide some preset motion models, adding the active range of motion practicing function, and adding visual feedback. The ease of use of the device operation receives an average score of 4.1. The therapist thinks it is easy to learn and operate, and the instruction is easy to follow.

The fourth subject is a PT having over ten years of clinical experience mainly working in the neurology and orthopedic fields. The robot operating experience is Reogo from Motorika and Hand of Hope from Rehab-Robotics. The safety of the device receives a score of 3.5. The therapist suggests optimizing the design of the connection bandage between the user’s arm and the robot arm, including increasing the width and decreasing the thickness to provide a more comfortable user experience for patients. The motion record function receives a score of 2 in clinical applicability. The therapist suggests adding the trajectory smoothing process and the speed-adjusting function. Regarding further optimization of the device’s function, the therapist suggests adding the assist training mode, the visual and audio feedback, and the training scheduling function. The ease of use of the device operation receives an average score of 3.3. The therapist thinks the gravity compensation process is too cumbersome and the process of changing sides takes too much time. The therapist suggests changing the design of the parts from screw type to bolt type to reduce the time spent.

The fifth subject is a PT having over ten years of clinical experience mainly working in the neurology and orthopedic fields. The robot operating experience is Reogo from Motorika and P100 from HIWIN Technology Corporation. The safety of the device receives a score of 3.5. The therapist suggests adding the function of assessing the range of motion of the user’s upper limb before training. The motion record function receives a score of 2 in clinical applicability since it is hard to record the demand motion. The therapist suggests adding the range of motion of the shoulder’s internal/external rotation and aligning the rotation axes of the user’s shoulder and the motor, providing the corresponding motion more accurately. The ease of use of the device operation receives an average score of 3.1. The therapist thinks the operation of changing sides and motion record function need some time to practice.

Therapists in the experience group give similar opinions on the device. The therapist is safe when operating the device, and the operating process is easy to learn. The motion record function should be optimized for proper clinical use. For now, the device cannot provide a smooth motion involving multiple joint movements since it is hard to record one. The therapists do not have a consistent opinion of the reason for the unsmooth recording; however, they all suggest adding the retouching process or option after recording. Recommendations for further optimization of the device mainly focus on developing the assist training mode (80%) and adding visual feedback (60%).

### 4.2. General Group

Twenty-nine therapists without robot operating experience were recruited for the general group.

#### 4.2.1. Safety

Regarding the safety issue, therapists gave average scores of 3.5. In the open-ended questions, feedback from therapists mainly focuses on environmental safety. There are six (21%) therapists who proposed adding functions of remote control and warning, and three therapists (10%) who proposed adding the function of locking the control panel to prevent the unintentional touch of others in the therapy room. Many of the hospitals in Taiwan place the rehabilitation robot in an independent room and allocate an exclusive therapist; therefore, the experiment sets the location of the situation in a room independent from other patients. In this situation, the robot rehabilitation course is usually not included in public health insurance. However, many of the therapists in the general group train patients who take the health insurance rehabilitation course in their daily work, and it usually takes place in an open therapy room. Since the development team does not limit the operating location of U100 for now, they should consider the operating situation in an open therapy room with other people, like patients and therapists, walking around. They should also consider the operating situation that the therapist may leave temporarily because therapists usually train multiple patients at the same time in the open therapy room.

#### 4.2.2. Function

In the functional topic, we ask therapists to assess the clinical applicability of the device and to give advice to optimize the design. The average scores of the function of the device are 2.3 for the motion record function and 4.0 for the gravity compensation function. Though the device does not claim to have the function of implementing functional training and tension releasing, we are asking therapists to express their opinion about using the device for these purposes to help the development team realize the designs demanded of these functions. The suggestions therapists give to provide functional training for the device mainly focus on the range of motion. Nine (31%) of the therapists suggest increasing the range of motion of shoulder internal rotation and external rotation, and five (17%) of them suggest opening the motion of the wrist and the hand by redesigning the interface between the distal arm and the device. As for the demanded designs of tension releasing, therapists suggest adding tension sensors and the function of recording pain-free range of motion.

Back to the main function of the device, which is the motion recording function, therapists give scores of 2.3 for clinical applicability. Summarizing the feedback in Table 4, the recommendations are sorted out into three different types (the reason for clinical inapplicability, the optimized orientation, and the implementation method), and the counts and the proportion of similar feedback are present in parentheses. Since there is no significant difference between the two groups, we discussed the feedback together. The main reason that lowers the clinical applicability is the replay trajectory of the recorded motion. Practicing motion with no smooth trajectory has no benefit for learning quality motions and may raise muscle tone.

Engineers usually evaluate the benefit and the cost to decide on the implementation, the orientation, and the method of modification. In this case, the benefit of the modification is avoiding inducing the muscle tone and avoiding causing negative effects on the training. The cost of modification is usually in accordance with the cost during the development process and the cost of mass production after the modification. The effect of training is the main function of U100; therefore, the benefit is enormous, and the modification of the recording function is imperative.

The purpose of the modification is to make sure that the replay trajectory is smooth. Therapists recommend two orientations to achieve the purpose, which is recording a smoother motion or retouching the replay trajectory. They provide some advice to optimize the function; however, some of the advice is partial in conflict or even contradiction. Sixteen therapists (46 percent) recommend adjusting the replay trajectory after recording. Most of the implementation methods they suggest are based on this orientation. To retouch the trajectory, therapists recommend updating functions like setting constant speed, setting maximum speed, adjusting speed and force manually, and previewing the trajectory. It shows that therapists think smoothness is related to the replaying speed of the trajectory.

The meaning of smoothness is continuity. In mathematics, the definition of smoothness is the continuity of the deviation of the function. The function may be a path of a certain movement, the trajectory of robot control, the planned route of auto vehicles, or even an audio track. In previous research, the measurements of smoothness in the motion analysis field are not consistent. Some of them defined it as the number of peak speeds during motion, some defined it as the mean speed divided by the peak speed (peak-speed ratio), while others defined it as normalized jerk [34,35]. The measurements of movement smoothness vary by sensorimotor, movements, and test requirements. However, only a few of them verified the validity and reliability of measurement methods. The log dimensionless jerk (LDLJ) and spectral arc length (SPARC) are two methods that verified validity, while SPARC verified its reliability, too [36]. According to the literature [32], the normal value of smoothness depends on tasks. The smoothness of different tasks should not be compared at the same level. What be determined in the previous work is that the movement of healthy subjects is smoother than subjects with neurological injury. The level of smoothness can be used as one of the references for recovery. Except for smoothness, the relationship between these kinematic parameters and the motor function of the upper limb has not been clearly defined. Research only shows that some of the parameters are moderate to high relative to clinical scales of upper limb motor function. The peak-speed ratio is high relative to the Motor Status score (MSS), the movement speed is highly relative to Fugl–Meyer score (FM), and the movement accuracy is highly relative to FM, MSS, and the Motor Power score (MP) [37].

**Figure 3:**
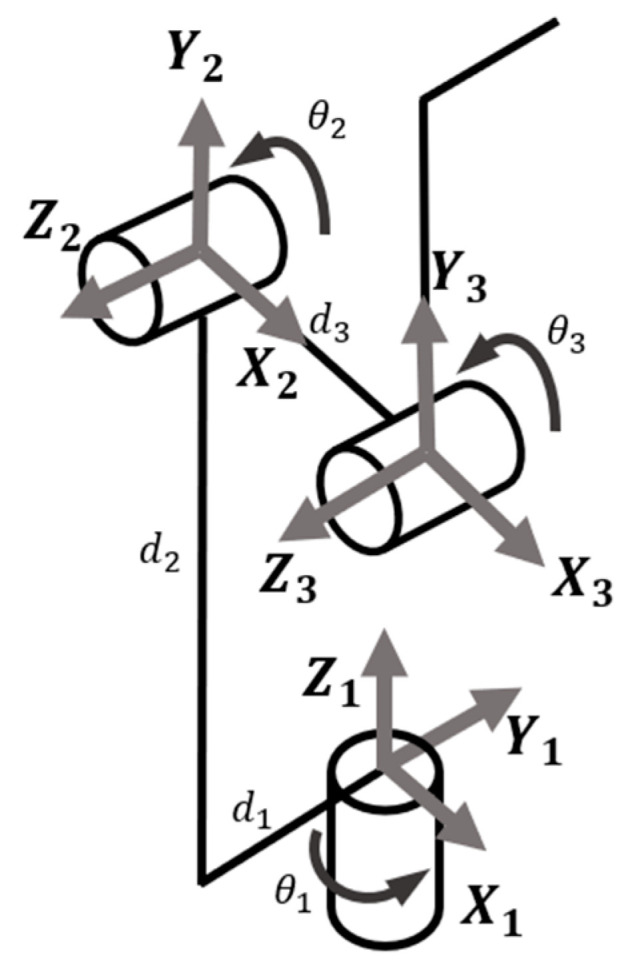
The coordinate frames of U100.

In the trajectory planning of a robot manipulator, smoothness is shown by the continuity of velocity and accelerator of the endpoint and the regular velocity of robot joints over time. Generally, when generating trajectories of the robot, the task points will first be confirmed, then a path passing all the task points will be generated, and finally, the trajectories will be generated after adding the trajectory constraints, like smoothness and structure limits. Finally, it may go through a partial smoothing process or use transition curves to smooth alone path segments. Trajectory smoothing applies interpolation and curves, including polynomials, linear function with parabolic blends, cubic splines, Bézier curve, asymptotic regression, etc., when generating trajectory. In the trajectory generation of U100, we first have the recorded joint movement. U100 records the angular position of motors over time in the recording process and replays it in the training. The robot arm position over time of the replay motion can be calculated by applying the direct kinematics process. After standardizing the coordinate frames of U100 (Figure 3), we can sort out the DH (Denavit–Hartenberg) table of U100 in Table 5. The angular position of motors over time can be transferred to the position over time of the robot arm. The velocity can then be calculated by differentiating the position to the base of the time. So far, we have the raw trajectory of the robot arm, and the next process is the partial smoothing of trajectories. Since the smoothness value of arm movement depends on tasks, and the main function of U100 emphasizes customization, which makes it hard to constrain the training movement, it is difficult to obtain a healthy smoothness level for each recorded motion. The partial trajectory smoothness process can only execute without constraint the smoothness level between different movements. According to the previous work, the 2D point-to-point movement from a healthy subject is in good agreement with the predicted minimum jerk model [30]. Other studies about partial trajectory smoothing are mainly applied to automated vehicle trajectory planning and obstacle avoidance [38,39,40,41,42]. Though the purpose of many of these trajectory-optimized methods is to reduce the execution time, they still provide a smooth trajectory. After smoothing the trajectory of the robot arm, we can calculate the demand angular position of the motors by applying an inverse kinematics process [43].

So far, the modification methods of retouching the trajectory can be preliminarily designed. The clinical applicability of the smoothed trajectory needs to be confirmed in the next formative test. Except for retouching the trajectory, we suggest the optimized robot providing the preview function and the manual adjusting function with the new trajectory. Since the retouched trajectory may not conform to the clinical needs, it may take many rounds of optimization to reach the goal. Through these two functions, engineers can collect the qualified trajectory as the reference for future designs. After recording and analyzing therapists’ choices of the option and the final smooth trajectory, the details of the recording function can then be consummated.

Although only five therapists (14 percent) recommend optimizing the recording procedure, since therapists mention the worry that the retouched trajectory may affect the training effect, this orientation cannot be neglected. Therapists recommend making the recording hand feel smoother and adjusting the friction of the exoskeleton joints. They think that the hand feelings are different between leading the exoskeleton and the patient’s arm, which causes an unsmooth trajectory. Manual therapy is widely used in stroke rehabilitation. Therapists are familiar with leading patients’ arms with specific control points and finely adjusted speed and force. Comparing the manual therapy and the operations of U100, there are several apparent differences. Visually, the control points and the structure of the exoskeleton and the human arm are different. U100 is recommended to be operated with both hands, with one hand controlling the upper arm linkage near the elbow joint and the other controlling the handle near the patient’s hand. The exoskeleton shoulder joints include three motors in different places on the exoskeleton, and the shoulder joint has three motion axes that intersect at the same point. Though the rotation axes of motors are close to human joints’ rotation axes, still there is a difference. Apart from visual differences, therapists also mention the friction of exoskeleton motors. They think the friction of the specific motor is too high and hinders them from recording a smooth trajectory. To simulate the feeling, the friction of motors can refer to the friction of human joints. The quantity of the friction of human joints can be measured, and the friction of motors can be calculated by robot kinematics [44,45,46].

However, the difference in friction cannot be regarded as the difference in hand feel. The relation between the device and the demanded hand feel is not clear, and the methods of optimization are discrete. It is hard to design the modification that surely is applicable in clinical settings since the design input is not clear. However, even if the device optimizes the recording function by updating the retouching function, it can be expected that therapists still have to try out and practice the leading process to record the suitable motion due to the difference. As a function in accordance with conventional therapy, the ideal statement is that therapists do not have to pay additional effort to know it well. The optimization of the recording process should also be emphasized. To find the clear design input for the optimized recording process, it can start by analyzing the difference between the raw recorded trajectory and the smoothed trajectory, finding the relation between intermittent points and robot designs, or the relation between leading the exoskeleton arm and the human arm.

#### 4.2.3. Ease of Use

In the ease-of-use issue, therapists from both groups gave an average score of 3.0. Therapists give scores for each operation process. The changing side process receives an average point of 2.4. The process of getting a patient on the device receives an average score of 2.8. The process of getting patients off the device receives an average score of 3.2. The process of wearing the connection band on the patient’s arm receives an average score of 3.1. The instructions for operating receive an average score of 3.4. Therapists’ feedback focuses on the operation of changing the training sides. They think the screw-type design takes too much time to operate (47%), and the device does not have clear instructions about how loose the screw type needs to be to be operated (44%). They suggest the development team change the design from screw type to bolt type (47%) to simplify the operation process and reduce the necessary operating time. The connection band is designed to be used in both the right and left hands, and there is only one size. Therapists suggest designing multiple sizes (41%) and marking the orientation of the band for wearing it faster (29%). It shows that therapists want the operation process to be simple, straightforward, and as fast as possible.

With the feedback of therapists in this study, future works for optimizing U100 can start by applying the smoothing process to the recorded trajectory and providing the preview function and manual adjustment function to obtain the details of the smooth trajectory. After providing a clinically applicable smooth trajectory, we suggest studying the difference between robot therapy executed by U100 and manual therapy and analyzing of interaction between the robot arm and the user’s arm. After studying the relations, U100 can assist the training more precisely and provide a great operating experience that only takes a short duration to practice. Except for providing a smooth replay trajectory, therapists recommend developing the assisted mode and active mode for patients with better motor function, since there is only a passive mode in U100 so far, and it is not suitable for providing challenging training for them. The active and assisted mode combined with the previous smoothness measurement function may also provide the referred criterion of motor function recovery. This article provides a pilot study about analyzing the user experience in a formative test and transforming the qualitative experience to the quantitative industrial specifications.

There are some limitations of this study that can be improved in future work. The trajectory of the robot and the kinematics of the human arm have not been recorded in this study; therefore, the trajectory difference between therapists cannot be analyzed. Reasons for the unsmooth trajectory are only described through the user experience of the therapist, lacking the analysis of the quantized statical data for a more specific discussion.

## 5. Conclusions

The safety of the novel rehabilitation device U100 is acceptable for the operators in this formative test. Since the participants in this study do not include real patients, further tests should be executed to confirm the safety. The operation process is easy to use. The main function is not clinically applicable due to the unsmooth replaying trajectory. We suggest starting the optimization by smoothing the trajectory after recording since the related methods are more specific than modifying the recording process. Further, a study about the clinical applicability of the smoothed trajectory after optimization should be executed.

## Figures and Tables

**Table 1:** Demographic statistics of recruited therapists. Main clinical experience field of PTs and OTs.

**PTs**	**General Group**	**Experience Group**
Neurological	8	1
Orthopedic	13	3
Cardiopulmonary	0	0
Pediatric	1	0
**OTs**	**General Group**	**Experience Group**
Physiological	6	1
Pediatric	2	0
Psychological	0	0

**Table 2:** The average score of questions on related topics in the questionnaire.

		Mean	Median	Stan.	*t*-Test
Safety	General	3.5	3.2	0.7	0.03
	Experience	4.5	5.0	0.6	
Clinical feasibility of motion record function	General	2.3	1.8	0.6	0.46
	Experience	2.4	2.0	0.2	
Ease of use	General	3.1	3.0	0.8	0.26
	Experience	3.6	3.7	0.7	

**Table 3:** Recommendations in open-ended questions about the record function.

Subject No.	Recommendations
1	It is hard to record a constant speed trajectory manually. Update the function that makes the speed constant.
2	Retouch the replaying trajectory.
3	The unsmooth trajectory could raise muscle tone. Retouch the replaying trajectory.
4	The unsmooth trajectory could raise muscle tone. Retouch the replaying trajectory.
5	Retouch the replaying trajectory. Used to lead patient’s arm with one hand in manual therapy, while the other stays on the shoulder to prevent hazards.
6	Retouch the replaying trajectory.
8	Optimize the recording process to lead more smoothly. The retouched trajectory may lose the effect of training.
9	Update the function of setting speed limits. Update the function of modifying the speed of trajectory after recording.
10	Retouch the replaying trajectory.
11	Cannot record a smooth motion trajectory.
12	Retouch the replaying trajectory.
13	Optimize the recording process to lead more smoothly. The friction in exoskeleton joints is too high to record a smooth motion.
14	Optimize the recording process to lead more smoothly or retouch the replaying trajectory.
15	Update the function that makes the speed constant.
16	Retouch the replaying trajectory or update the function that makes the speed constant.
17	Cannot record a smooth motion trajectory since the joints of exoskeleton action are separated. Recommend optimizing the recording process to lead more smoothly to avoid the motion trajectory being modified.
18	Recommend optimizing the recording process to lead more smoothly to avoid the motion trajectory being modified. Cannot record a smooth motion trajectory since the joints of exoskeleton action are separated.
19	Update the function to preview the recorded trajectory. The function of adjusting replaying motion force.
21	Retouch the replaying trajectory. Setting the interval between trials.
22	Cannot record a smooth motion trajectory at low speed.
23	Update the function of retouching the replaying trajectory or setting the replaying speed.
24	Retouch the replaying trajectory or update the function that makes the speed constant.
25	Retouch the replaying trajectory or set the replaying speed.
26	Cannot record the motion like leading human arm since the structure is different.
27	Cannot record the motion like leading human arm since the structure is different.
28	Cannot record a smooth motion trajectory due to the feedback of force of exoskeleton joints.
29	Cannot record a smooth motion trajectory due to exoskeleton joints.
31	Cannot record a smooth motion trajectory involving multiple joints.
32	Cannot record a smooth motion trajectory involving multiple joints. The friction of the supination/pronation joint is too high.
33	Provide several motion options to choose.
34	Update the function to adjust the recorded speed. The friction of the supination/pronation joint of the exoskeleton is too high.

**Table 4:** Summary of modifying recommendations.

	Description (Count/Proportion) among Two Groups
Reason	Unsmooth replay trajectory (31/89%)
Purpose	Make the trajectory smoother
Orientation	Adjust the trajectory after recording (16/46%) Optimize the recording process (5/14%)
Methods	Retouch the trajectory after recording (13/37%) Retouch the replaying speed constantly (4/11%) Setting speed limitation (1/3%) Adjust motors’ friction (3/9%)
Other feedback	Update the preview function Update the function to set the interval between trials

**Table 5:** The DH table of U100.

i	$\alpha_{i-1}$	$a_{i-1}$	$d_{i}$	$\theta_{i}$
1	0	0	0	$\theta_{1}$
2	$90^{{}^{\circ}}$	0	$\sqrt{d_{1}^{2}+d_{2}^{2}}$	$\theta_{2}$
3	0	$d_{3}$	$d_{3}$	$\theta_{3}$

## Data Availability

The data that support the findings of this study are available from the corresponding author, upon reasonable request.

## Supplementary Materials

The following supporting information can be downloaded at https://www.mdpi.com/article/10.3390/s23219003/s1. Two types of questionnaires: multiple-choice and open-ended questions.

## References

[1] Cause of Death Statistics-Ministry of Health and Welfare. https://dep.mohw.gov.tw/dos/np-1776-113.html.

[2] Mohr J., Wolf P.A., Moskowitz M.A., Mayberg M.R., Von Kummer R. (2011). Stroke E-Book: Pathophysiology, Diagnosis, and Management.

[3] McCabe J., Monkiewicz M., Holcomb J., Pundik S., Daly J.J. (2015). Comparison of robotics, functional electrical stimulation, and motor learning methods for treatment of persistent upper extremity dysfunction after stroke: A randomized controlled trial. Arch. Phys. Med. Rehabil..

[4] Mehrholz J., Hädrich A., Platz T., Kugler J., Pohl M. (2012). Electromechanical and robot-assisted arm training for improving generic activities of daily living, arm function, and arm muscle strength after stroke. Cochrane Database Syst. Rev..

[5] Rozevink S.G., Hijmans J.M., Horstink K.A., van der Sluis C.K. (2021). Effectiveness of task-specific training using assistive devices and task-specific usual care on upper limb performance after stroke: A systematic review and meta-analysis. Disabil. Rehabil. Assist. Technol..

[6] Everard G., Declerck L., Detrembleur C., Leonard S., Bower G., Dehem S., Lejeune T. (2022). New technologies promoting active upper limb rehabilitation after stroke: An overview and network meta-analysis. Eur. J. Phys. Rehabil. Med..

[7] Yeh T.-N., Chou L.-W. (2022). Clinical Demands of Designs for Rehabilitation Robots in Taiwan. Innovation.

[8] Housman S.J., Scott K.M., Reinkensmeyer D.J. (2009). A randomized controlled trial of gravity-supported, computer-enhanced arm exercise for individuals with severe hemiparesis. Neurorehabilit. Neural Repair.

[9] Oña E., Cano-de La Cuerda R., Sánchez-Herrera P., Balaguer C., Jardón A. (2018). A review of robotics in neurorehabilitation: Towards an automated process for upper limb. J. Healthc. Eng..

[10] Sale P., Lombardi V., Franceschini M. (2012). Hand robotics rehabilitation: Feasibility and preliminary results of a robotic treatment in patients with hemiparesis. Stroke Res. Treat..

[11] Nef T., Guidali M., Klamroth-Marganska V., Riener R. (2009). ARMin-exoskeleton robot for stroke rehabilitation. Proceedings of the World Congress on Medical Physics and Biomedical Engineering.

[12] Bovolenta F., Sale P., Dall’Armi V., Clerici P., Franceschini M. (2011). Robot-aided therapy for upper limbs in patients with stroke-related lesions. Brief report of a clinical experience. J. Neuroeng. Rehabil..

[13] Lee S.H., Park G., Cho D.Y., Kim H.Y., Lee J.-Y., Kim S., Park S.-B., Shin J.-H. (2020). Comparisons between end-effector and exoskeleton rehabilitation robots regarding upper extremity function among chronic stroke patients with moderate-to-severe upper limb impairment. Sci. Rep..

[14] Moggio L., de Sire A., Marotta N., Demeco A., Ammendolia A. (2022). Exoskeleton versus end-effector robot-assisted therapy for finger-hand motor recovery in stroke survivors: Systematic review and meta-analysis. Top. Stroke Rehabil..

[15] Lo A.C., Guarino P.D., Richards L.G., Haselkorn J.K., Wittenberg G.F., Federman D.G., Ringer R.J., Wagner T.H., Krebs H.I., Volpe B.T. (2010). Robot-assisted therapy for long-term upper-limb impairment after stroke. N. Engl. J. Med..

[16] Gijbels D., Lamers I., Kerkhofs L., Alders G., Knippenberg E., Feys P. (2011). The Armeo Spring as training tool to improve upper limb functionality in multiple sclerosis: A pilot study. J. Neuroeng. Rehabil..

[17] Nef T., Guidali M., Riener R. (2009). ARMin III–arm therapy exoskeleton with an ergonomic shoulder actuation. Appl. Bionics Biomech..

[18] Wolbrecht E.T., Chan V., Reinkensmeyer D.J., Bobrow J.E. (2008). Optimizing compliant, model-based robotic assistance to promote neurorehabilitation. IEEE Trans. Neural Syst. Rehabil. Eng..

[19] Ata A.A. (2007). Optimal trajectory planning of manipulators: A review. J. Eng. Sci. Technol..

[20] Gasparetto A., Boscariol P., Lanzutti A., Vidoni R. (2015). Path planning and trajectory planning algorithms: A general overview. Motion and Operation Planning of Robotic Systems: Background and Practical Approaches.

[21] Faroni M., Beschi M., Visioli A., Pedrocchi N. (2021). A real-time trajectory planning method for enhanced path-tracking performance of serial manipulators. Mech. Mach. Theory.

[22] Cheng J., Chen Y., Zhang Q., Gan L., Liu C., Liu M. Real-time trajectory planning for autonomous driving with gaussian process and incremental refinement. Proceedings of the 2022 International Conference on Robotics and Automation (ICRA).

[23] Guo D., Xu F., Yan L., Nie Z., Shao H. (2018). A new noise-tolerant obstacle avoidance scheme for motion planning of redundant robot manipulators. Front. Neurorobot..

[24] Jin L., Su Z., Fu D., Xiao X. (2023). Coevolutionary Neural Solution for Nonconvex Optimization With Noise Tolerance. IEEE Trans. Neural Netw. Learn. Syst..

[25] Hentout A., Maoudj A., Aouache M. (2023). A review of the literature on fuzzy-logic approaches for collision-free path planning of manipulator robots. Artif. Intell. Rev..

[26] Xie Z., Zhang Q., Jiang Z., Liu H. (2020). Robot learning from demonstration for path planning: A review. Sci. China Technol. Sci..

[27] Wiklund P.M.E., Kendler J., Strochlic A.Y. (2015). Usability Testing of Medical Devices.

[28] Liu S.-E., Hsieh F.-H. (2021). Exoskeleton Apparatus for Limb Rehabilitation. U.S. Patent.

[29] Hsieh F.-H., Huang Y.-W. (2021). Upper Limb Training System and Control Method Thereof. U.S. Patent.

[30] McCallum S., Milner M.M. (2021). The effectiveness of formative assessment: Student views and staff reflections. Assess. Eval. High. Educ..

[31] Umer M., Javid C.Z., Farooq M.U. (2013). Formative assessment: Learners’ preferred assessment tasks, learning strategies and learning materials. Kashmir J. Lang. Res..

[32] Ragupathi K. (2020). Gathering Formative Feedback Through Mid-Semester Evaluations.

[33] Shabana A., Humaira Fayyaz K. (2016). Impact of combined modular assessment on deep learning and personal development of medical students. Pak. J. Med. Sci..

[34] Goffredo M., Mazzoleni S., Gison A., Infarinato F., Pournajaf S., Galafate D., Agosti M., Posteraro F., Franceschini M. (2019). Kinematic parameters for tracking patient progress during upper limb robot-assisted rehabilitation: An observational study on subacute stroke subjects. Appl. Bionics Biomech..

[35] Schwarz A., Kanzler C.M., Lambercy O., Luft A.R., Veerbeek J.M. (2019). Systematic review on kinematic assessments of upper limb movements after stroke. Stroke.

[36] Balasubramanian S., Melendez-Calderon A., Roby-Brami A., Burdet E. (2015). On the analysis of movement smoothness. J. Neuroeng. Rehabil..

[37] Do Tran V., Dario P., Mazzoleni S. (2018). Kinematic measures for upper limb robot-assisted therapy following stroke and correlations with clinical outcome measures: A review. Med. Eng. Phys..

[38] Flash T., Hogan N. (1985). The coordination of arm movements: An experimentally confirmed mathematical model. J. Neurosci..

[39] Garber M., Lin M.C. (2004). Constraint-based motion planning using voronoi diagrams. Algorithmic Foundations of Robotics V.

[40] Park C., Pan J., Manocha D. ITOMP: Incremental trajectory optimization for real-time replanning in dynamic environments. Proceedings of the International Conference on Automated Planning and Scheduling.

[41] Rösmann C., Hoffmann F., Bertram T. Kinodynamic trajectory optimization and control for car-like robots. Proceedings of the 2017 IEEE/RSJ International Conference on Intelligent Robots and Systems (IROS).

[42] Ravankar A., Ravankar A.A., Kobayashi Y., Hoshino Y., Peng C.-C. (2018). Path smoothing techniques in robot navigation: State-of-the-art, current and future challenges. Sensors.

[43] Li G., Fang Q., Xu T., Zhao J., Cai H., Zhu Y. (2019). Inverse kinematic analysis and trajectory planning of a modular upper limb rehabilitation exoskeleton. Technol. Health Care.

[44] Fujisawa T., Takagi M., Takahashi Y., Inoue K., Terada T., Kawakami Y., Komeda T. Basic research on the upper limb patient simulator. Proceedings of the 2007 IEEE 10th International Conference on Rehabilitation Robotics.

[45] Cz N.A., Komeda T., Low C.Y. (2012). Design of upper limb patient simulator. Procedia Eng..

[46] Takhashi Y., Komeda T., Koyama H., Yamamoto S.-I., Arimatsu T., Kawakami Y., Inoue K., Ito Y. Development of an upper limb patient simulator for physical therapy exercise. Proceedings of the 2011 IEEE International Conference on Rehabilitation Robotics.

