# Supplementary material for: User Experience Evaluation of Upper Limb Rehabilitation Robots: Implications for Design Optimization: A Pilot Study
Source: Sensors (Basel). 2023 Nov 6;23(21):9003. doi: 10.3390/s23219003 (PMC10647564; doi:10.3390/s23219003)
Supplement: Supplementary file 1 [file sensors-23-09003-s001.zip › sensors-2561668-supplementary.pdf]

Supplementary material:

Two types of questionnaires: multiple-choice and open-ended questions.

Part I demographic statistics

1. How many years have you worked in this region?

□1~3 □3~5 □5~10 □over10 the years

2. Are you a PT or OT?

□PT (Please continue to answer question 3)

□OT (Please continue to answer question 4)

3. Please sort these fields by your clinical experience quantity. (Please fill 1 ~ 4 for more to less)

_Neurological _Orthopedic _Pediatric _Cardiopulmonary

4. Please sort these fields by your clinical experience quantity. (Please fill 1 to 3 for more to less)

_Physiological _Psychological _Pediatric

Part II survey about clinical applicability: rating scale item

Exterior and portability

1. How do you feel about this robot?

□unsteady □sometimes unsteady □steady, but I'll still worry □steady □very steady

2. How do you feel about the portability of this robot?

□It's hard to move □Sometimes it's hard to move. □I can move it, but still need practice □It's ease to move □Very portable

3. How do you feel about the exterior of this robot? □ugly □unattractive □average □good □beautiful

4. How do you feel about time spent on correction?

□too long □long □nothing □shorter than expect □quite fast

5. How do you feel about equipping the assistance equipment on a patient's limb?

□hard to operate □need some practice □easy □easy, and do not need any practice □ease to use, and fast

6. How do you feel about time spent on getting patient on the robot?

□too long □long □nothing □shorter than expect □quite fast

7. How do you feel about operating procedures that get patients on the robot?

□hard to operate □need some practice □easy □easy, and do not need any practice □ease to use, and fast

8. How do you feel about getting the patient off the robot?

□hard to operate □need some practice □easy □easy, and do not need any practice □ease to use, and fast

9. How do you feel about the procedures changing the effect side of the robot?

□hard to operate □need some practice □easy □easy, and do not need any practice □ease to use, and fast

10. How do you feel about the operating procedures of this robot?

□hard to operate □need some practice □easy □easy, and do not need any practice □ease to use, and fast

11. How do you feel about operating instructions on the screen?

□hard to understand □usually confuse □sometimes confused □I can understand it without too much thinking □easy to understand

12. How do you feel about the touch screen?

□always lag □a little bit lag □nothing □sensitive □ease to use

13. How do you feel about the troubleshooting procedures?

□hard to operate □need some practice □easy □easy, and do not need any practice □ease to use, and fast

14. How do you feel about troubleshooting instructions on the screen?

□hard to understand □usually confuse □sometimes confused □I can understand it without too much thinking □easy to understand

15. How do you feel about the instructions reminding changing procedures?

□hard to understand □usually confuse □sometimes confused □I can understand it without too much thinking □easy to understand

16. How do you feel about the instructions for emergency stop?

□hard to understand □usually confuse □sometimes confused □I can understand it without too much thinking □easy to understand

17. How do you feel about using the robot for training drinking activities?

□conventional therapy is better □similar to conventional therapy, but still not enough □having the same effect as conventional therapy □similar to conventional therapy, and slightly better □way better than conventional therapy

18. What activities will you choose to train skills of drinking in conventional therapy?

□PROM □PNF □shoulder arc □ring tree □nuts and bolts □cups pass □gripping □insert board □claymore

19. How do you feel about using the robot for training dressing activities?

□conventional therapy is better □similar to conventional therapy, but still not enough □ has the same effect as conventional therapy □similar to conventional therapy, and slightly better □way better than conventional therapy

20. What activities will you choose to train skills of drinking in conventional therapy?

□PROM □PNF □shoulder arc □ring tree □nuts and bolts □cups pass □gripping □insert board □claymore

21. How do you feel about using the robot to record motions of releasing tension?

□not applicable □need some practice □nothing □applicable □ease to use

22. How do you feel about using the robot to release tension?

□having the same effect as conventional therapy □similar to conventional therapy, and slightly better □way better than conventional therapy

23. How do you feel about allowing record time?

□too short □a bit short □nothing □enough in most conditions □enough

24. How do you feel about the replaying trajectories of robots?

□totally different from the recorded motion □a bit different from the recorded motion, and may drop the training effect □a bit different from the recorded motion, but acceptable □exactly replaying recorded motion, but a little more modifying assistance will be helpful □exactly replaying motions that I had in mind

25. How do you feel about the trajectories of the patient's limb led by the robot?

□totally different from the recorded motion □a bit different from the recorded motion, and may drop the training effect □a bit different from the recorded motion, but acceptable □exactly replaying recorded motion, but a little more modifying assistance will be helpful □exactly replaying motions that I had in mind

26. How do you feel about the gravity compensation support of the robot?

□it makes training harder than the conventional way □better not having the support □feeling nothing different with this function □it can ease my workload □very helpful

27. Is the record-replay function applicable in clinical?

□unapplicable □conceptually applicable, but still need to improve for actual use □applicable □applicable, and developmental □applicable, and has high development prospect

28. Do you feel safe when getting the patient on the robot?

□risk of hazard □not safe □safe, but need to pay attention to robot's movement □safe □very safe

29. Do you feel safe while training?

□risk of hazard □not safe □safe, but need to pay attention to robot's movement □safe □very safe

30. Do you feel safe while correction?

□risk of hazard □not safe □safe, but need to pay attention to robot's movement □safe □very safe

Part III: open-end question

1. Do you have any options about questions in the second part?

2. Do you have any suggestions for the development and improvement of the robot?
